# Diverse Microbiota Identified in Whole Intact Nest Chambers of the Red Mason Bee *Osmia bicornis* (Linnaeus 1758)

**DOI:** 10.1371/journal.pone.0078296

**Published:** 2013-10-18

**Authors:** Alexander Keller, Gudrun Grimmer, Ingolf Steffan-Dewenter

**Affiliations:** 1 Department of Animal Ecology and Tropical Biology, Biocenter, University of Wuerzburg, Wuerzburg, Germany; 2 DNA Analytics Core Facility, Biocenter, University of Wuerzburg, Wuerzburg, Germany; Ghent University, Belgium

## Abstract

Microbial activity is known to have profound impact on bee ecology and physiology, both by beneficial and pathogenic effects. Most information about such associations is available for colony-building organisms, and especially the honey bee. There, active manipulations through worker bees result in a restricted diversity of microbes present within the colony environment. Microbial diversity in solitary bee nests remains unstudied, although their larvae face a very different situation compared with social bees by growing up in isolated compartments. Here, we assessed the microbiota present in nests and pre-adults of *Osmia bicornis*, the red mason bee, by culture-independent pyrosequencing. We found high bacterial diversity not comparable with honey bee colonies. We identified a variety of bacteria potentially with positive or negative interactions for bee larvae. However, most of the other diverse bacteria present in the nests seem to originate from environmental sources through incorporated nest building material and stored pollen. This diversity of microorganisms may cause severe larval mortality and require specific physiological or symbiotic adaptations against microbial threats. They may however also profit from such a diverse environment through gain of mutualistic partners. We conclude that further studies of microbiota interaction in solitary bees will improve the understanding of fitness components and populations dynamics.

## Introduction

Beekeepers early identified the impact of bacterial organisms on honey bees, making pathological analyses and serological cultures important tools to assess hive diseases and oncoming threats [1]. Honey-bees are very susceptible to some bacteria, as exemplified by the American and European foulbrood caused by *Paenibacillus larvae* subsp. *larvae* and *Melissococcus plutonius*, respectively. Other bacteria have been reported to accompany pathogenic agents and increase their virulency [2]. However, there are not only negative effects mediated by bacteria, although the major part of investigations concentrates on disease-related organisms. Likewise to other animals, consortia of bacteria also associate as part of natural microbial communities with neutral or positive effects on the bee host [3], yet studies about non-pathogenic microbes are still in their infancy and mostly restricted to gut bacteria [1,4-11].

Especially little is known in this regard about non-hive bees and their nests, although such associations are also expectable. The organism of interest in this study, *Osmia bicornis* (Megachilidae), is a species of solitary bees native to Europe and Northern Africa. It inhabits natural as well as anthropogenically altered environments. It is a very efficient pollinator with high pollination rates due to very frequent stigmata contacts during gathering of pollen [12-15]. Females build their nests in existing hollow spaces and place their eggs alongside collected pollens. Each brood cell with stored pollen and an individual egg is separated with a loam wall and the nest entrance is also closed with loam. This results in several chambers following each other so that each harbors only a single egg. Hatching occurs after approximately a week, and development of the larvae takes place until late summer. Subsequent they spin cocoons and enter the pupal stage. Fully developed adults hibernate within cocoons and leave their nests in spring. As female-destined eggs are laid in the inner chambers, males emerge first and wait for copulation. Although *Osmia bicornis* is a widespread and well investigated solitary bee species that shows high levels of unexplained larval mortality [16], for bacteria neither pathogens nor mutualistic symbionts have been identified so far [17]. 

Thus, most of the available information about bee associated bacteria originates from studies with honey and bumble bees. The body surface of adult honey bees is relatively free of bacteria, likely due to grooming behavior [1]. The set of bacteria in an adult honey-bee gut is low in diversity and typically described to be composed of only eight different taxa [1,3,5]. Similar to that, bumble-bee gut microbiota have been described as very distinct and sparse in diversity [8]. It is currently controversial whether variation according to biogeography of the hives exists [1,3,8,9,18,19]. 

Several bacteria are also suspected to be involved in the bioconversion and preservation of pollen material [6,20]. They are actively secreted by worker bees onto newly collected pollen grains. Subsequently these bacteria reduce the diversity of other microbial organisms that were originally present. Nectar and honey themselves have native antimicrobial properties. These components and pollen-derived jelly compose the primary food source to honey-bee offspring and prevent establishment of microorganisms in larvae [21]. Resin as an well used component of honey bee hives shows also antimicrobial properties [22]. It has thus been suggested that honey bee pre-adults are almost sterile systems [23-25] and inoculation with intestinal bacteria occurs after hatching of adults [26]. However, some newer studies were able to recognize bacteria also within guts of larvae [9,11], which assembled a different community than those found in adults, referable to their alternate nutrition.

There are some indications that these associations are evolutionary well conserved. Inoculation reports were made for honey-bees and also stingless bees feeding on pollen, but interestingly also for dead animal tissue collected by the necrophagic bee *Trigona hypogea*. It has been suggested, that this is a potentially very ancient symbiosis after a finding of bees and bacteria together enclosed in amber [27]. Beside gut bacteria and those involved in food preparation, honey bees seem to foster establishment of few other bacteria with antimicrobiotic or antimycotic capabilities within their hives [28]. Microbial associates are thus important components of a functional colony system. 

Despite an ancient history and conservation of symbiotic associations, we however expect differences between honey bees and solitary bees according to their social or non-social way of life. Especially offspring of solitary bees face a very different situation from hive bees, i.e. depending on pollen for their development rather than pre-manipulated jellies. Further, larvae do not develop in a constantly tended environment and are not actively supported by nurses [24,29,30]. It is thus of great importance to assess their microbial ecology and identify patterns alike or different from hive-bees.

In this study we investigated whole nests including almost fully developed pupae of the red mason-bee *O. bicornis* through cultivation-independent next-generation sequencing to identify accompanying bacteria and their multiple possible origins. We aim to provide an initial assessment of the microbiota associated to a solitary bee nest and to gain first insights into its differences and similarities with those of honey bee hives. 

## Methods

### Sampling

A reed stem containing a *Osmia bicornis* nest with brood cells was taken from an artificial stack at a grassland site near the Biocenter of the University of Würzburg, Germany (Latitude 49° 46' 47.78, Longitude 9° 58' 22.55) in October 2012, few weeks before hibernation of *Osmia* was initiated. The experimental site was property of the University of Würzburg and is regularly used for behavioral and ecological studies on Hymenoptera. The reed was split in half lengthwise and revealed four nest chambers with each an adequately developed pre-adult present and surrounded by an intact cocoon. Pupae including cocoons weighted between 90 mg and 140 mg in total. For our analyses, we used the first and the last chamber, numbered chamber C1 and C4 according to the reed-exit position, with C1 identifying the outward bound chamber. Directly after C4 a reed node followed. C1 and C4 were thus the newest and oldest chamber harboring most likely a female and a male, respectively, and were treated separately as two individual samples in the following. Chambers were not visibly affected by macro-pathogens, parasites or nest destroyers. As far as observable, they were intact and the individuals were healthy. 

### DNA extraction

For each chamber, we sampled all nest contents combined (including bees, cocoon, remaining pollen) for DNA extraction. Further swabs of the complete interior i.e. chamber walls and loam barriers were taken with sterile cutton buds. The merged pools of chamber contents were extracted with a spatula and together with the swabs transferred into the kit lysis buffer. We used the MoBio PowerSoil DNA Isolation kit (Carlsbad, CA, USA), adequate for microbial DNA extraction in environmental samples. The substance in the buffer was mechanically disrupted with an electric hand-held homogenizer. The following extraction and isolation steps were performed according to the manufacturers instructions. The extraction kit uses a silica bead-beating step, which was performed on a vortexer with self-made horizontal probe mounts for 10 minutes at maximum speed. All tasks were performed with gloves and our tools were sterilized with 70% ethanol between sampling steps. 

### Amplicons

For PCR amplification, we used primers to amplify 16S ribsomal DNA of bacteria according to Hamady et al. [31] that enclose the variable regions V1-V2. It was found to be well suited for the phylogenetic analysis of pyrosequencing reads [31-34]. We used “fusion” primers designed to have 454 adapter regions and the targeting primers. The forward “fusion” primer (5’-CGTATCGCCTCCCTCGCGCCA-TCAG-AGAGTTTGATCCTGGCTCAG-3’) consists of the 454 specific Adapter A, the linker key and the conserved forward primer 27f (the corresponding regions are delineated in the sequence by hyphens). The reverse “fusion” primer (5’-CTATGCGCCTTGCCAGCCCGC-TCAG-XXXXXXXXXX-CATGCTGCCTCCCGTAGGAGT-3’) contains the 454 specific Adapter B, the linker key, an multiplex identifier (MID) and the bacterial primer 338f (regions delineated in the sequence by hyphens). MIDs were used to analyze several samples together on the same sequencing chip. For our two nest samples we used the MIDs 5 (ATCAGACACG) and 7 (CGTGTCTCTA) officially suggested by Roche in a technical bulletin (454 Sequencing Technical Bulletin No. 005-2009, April 2009). Their position is indicated by placeholders “X” in the aforementioned reverse “fusion” primer sequence. The remaining MIDs were used for different projects using the same primers and target organisms (bacteria). Fusion primers were constructed at the Metabion laboratories (Martinsried, Germany).

PCR reaction mixes consisted of 0.25 µl of each forward and reverse primer (each 30µM molar), 3 µl of template DNA and 25µl of Phusion High-Fidelity DNA polymerase PCR 2x MasterMix (Thermo Scientific, Waltham, MA 02454, USA). Bidest H_2_O was added to a reaction volume of 50 µl. Samples were initially denaturated at 98 °C for 30 s, then amplified by using 35 cycles of 98 °C for 10 s, 50 °C for 30 s and 72 °C for 30 s. A final extension (72 °C) of 10 min was added at the end of the program to ensure complete amplification. 

All samples were amplified in ten separate aliquots to reduce random effects on the community during PCR. PCR amplicons of these ten replicates were combined, gel-electrophoresed, trimmed for amplicon length and cleaned with the HiYield PCR Clean-up Kit (Real Biotech Corporation, Banqiao City, Taiwan) according to the manufacturers description. Cleaned samples were quantified using a Qubit II Flurometer (Invitrogen/Life Technologies, Carlsbad, CA, USA) and the dsDNA High-Sensitivity Assay Kit (also Invitrogen/Life Technologies) as described in the vendors protocol. We used the BioAnalyzer 2200 (Agilent, Santa Clara, CA, USA) with High Sensitivity DNA Chips (also Agilent) for verification of fragment length distributions.

### Library preparation and sequencing

Pyrosequencing and library preparation was performed according to the guidelines for the GS FLX junior (Roche, Basel, Switzerland). Samples were diluted to 1x10^9^ molecules/µl in 1x TE buffer. For multiplexed pyrosequencing, 10 individual samples were pooled in equal amounts as an amplicon pool. This pool was afterwards again diluted to a concentration of 1x10^7^ molecules/µl with molecular biology grade water. Emulsions were prepared on the IKA Ultra Turrax Tube Drive and later aliquoted into 96 well plates. The following emPCR was performed according to the guidelines for 454 sequencing by Roche including bead recovery, enrichment and bead count steps using Roche GS junior (Roche, Basel, Switzerland) equipment and consumables. Sequencing was performed in-house with a GS junior device located in the Department of Human Genetics (University of Würzburg, Germany) with original Roche GS junior titanium chemistry.

### Filtering and data quality assessment

Data was demultiplexed into the different samples using the MID adapter sequences and the QIIME software [35,36]. During this step, only sequences spanning both priming regions were further used, i.e. only completely sequenced amplicons. Primers, adapters and MIDs were trimmed. Chimeric checking and quality filtering was as well performed during this step. We restricted data only to high quality reads with a phred score ≥ 27 [37], and no reads with ambiguous characters or homopolymers exceeding five bases were included in the following downstream analyses. 

Prior to diversity and abundance estimations deduplication of identical sequences reduce artificial amplification biases and only allow nearly identical sequences to be considered for the measures [38]. This step was performed with CD Hit 454 [38], thus further analyses were made with unique reads. 

All unique reads were assigned to taxonomic clades by using the *RDPclassifier* [39,40]. The classifier assigns taxonomic ranks as deep as possible with estimated certainty by using a Bayesian approach. We set the threshold to a 50% bootstrap cutoff for ranks to be adapted, which has been shown to assign more than 90% of sequences to genus level with 95% accuracy in most variable regions of the 16S. We restricted our estimations to the genus level to reduce artifical overestimation of taxonomic units, which is stringent below 97% clustering as suggested by Kunin et al. [37]. This ensures that analyses provide accurate profiling of microbial communities [37]. Singletons were excluded from further analyses.

Chloroplast reads were considered to be contamination due to nest-building material and the reed themselves and accordingly removed. Also, pollen was reported to carry occasional plastid genomic DNA [41]. Hierarchic taxonomic assignments for all bacteria on a generic level were displayed and investigated with KronaTools [42]. 

The other samples processed with the same sequencing chip included 16S microbial samples obtained non-invasively from surfaces of invertebrate animals that were cultured according to Schokraie et al. [43](3) and plants (5). We assumed the origins to be very different and unlikely to share large proportions of the microbiota. Omnipresent species were thus considered as lab contamination and ignored in the following analyses. 

Raw sequencing data alongside quality information was uploaded to the public database for environmental sequencing data of the European Nucleotide Archive (EMBL-EBI: ENA) and are retainable through the SRA study accession number ERP002613.

Specific bacteria of interest, i.e. pathogens or commensals known to be of importance for bees or other arthropods were identified to species level by BLASTn [44]. These clades specifically screened for were [1,9,45-59]:

• Gut bacteria: *Bacillus subtilis* (strains C4, G2III and M1), *Bartonella, Bifidobacterium*, *Burkholderia cepacia, Gilliamella*, *Enterobacter, Gluconobacter*, *Klebsiella, Lactobacillus, Saccharibacter, Snodgrassella.*
• Potential pathogens: *Achromobacter*, *Bacillus cereus, Bacillus thuringiensis, Brevibacillus*, *Clostridium botulinum, Enterococcus*, *Melissococcus plutonius*, *Mesoplasma*, *Paenibacillus*, *Photorhabdus luminescens*, *Pseudomonas entomophila, Pseudomonas protegens*, *Rickettsiella grylli, Spiroplasma melliferum, Xenorhabdus bovienii, Xenorhabdus nematophila, Stenotrophomonas.*
• Non-pathogenic intracellular bacteria: *Mycoplasma, Regiella insecticola*, *Rickettsia*, *Sulcia, Wolbachia, Zinderia.*
• Associated with pollens: *Aurobasidium, Bacillus, Rhizopus.*

For each of these taxa of interest, we prepared a local BLAST database populated with all 16S sequences of this group present at NCBI (accession date 10th February 2013). We aligned all of our RDP clade-classified sequences against the corresponding databases. The resulting local pairwise alignments were accepted if longer than 250bp. The best hit according to sequence identity was chosen as representative. Pyrosequencing reads are known to tend to overestimation of biodiversity, thus we chose our threshold in concordance with Kunin et al. [37]. Hits with identities below 90% were discarded, 90-94% treated as closely related organisms, 95-97% as likely same species but different subspecies/strains and lastly with more than 97% declared as the same species, subspecies and strain. 

## Results and Discussion

### Sequencing results

Two nest chambers including bees of an artificial reed stack containing *Osmia bicornis* were investigated through pyrosequencing for their bacterial communities. The total sequencing chip (including eight samples for other studies) yielded 40.684 reads and 13,4 Mbp passing Roche’s GS Run Browser quality filtering step. Of these, 36.167 sequences were assignable to their multiplex origin. After demultiplexing and further manual filtering (chimeras, ambiguous positions, homopolymers, missing primers, phred score), we received a total of 7.925 16S sequences dedicated to this study, with 4797 and 3128 reads respectively for chambers C1 and C4. After removal of chloroplast reads and identical sequences (as generated through PCR amplification), we obtained 2668 deduplicated unique bacterial sequences. 

### Bacterial diversity and community composition

**Figure 1:**
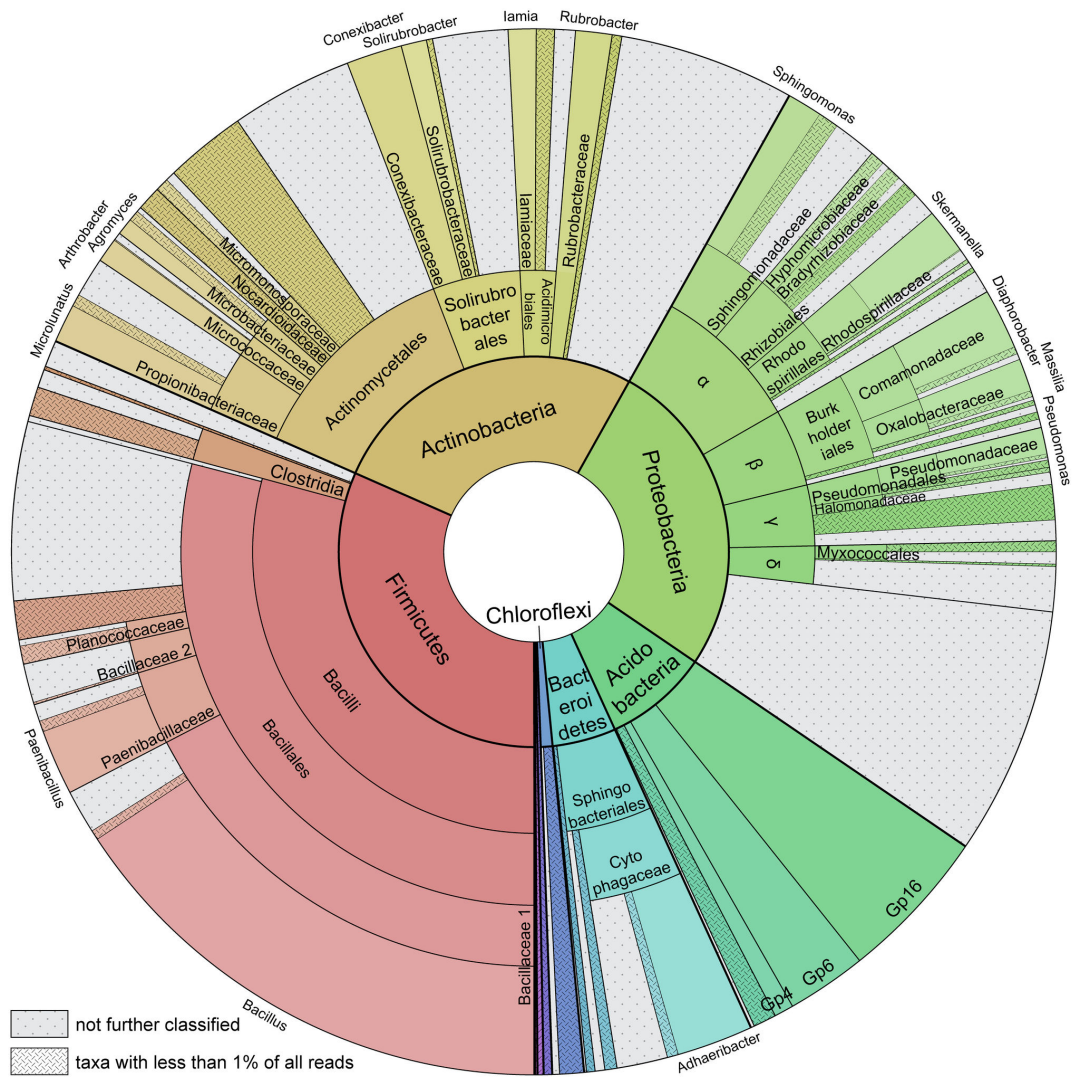
Taxonomic distribution of the microbiota according to read classification in both chambers. Classification is according to the RDP classifier with 0.8 bootstrap cutoff. Reads unassignable at the generic level were included as far as possible in the hierarchical lineage and are displayed with dots. Taxonomic groups with less than 1% share of total number of reads were combined for the sake of clarity and are illustrated by crossed stripes.

The composition of taxonomic groups was very similar between the two samples, including the division of reads into families within the major clades (Tab. 1). Most dominant groups were the Proteobacteria, Firmicutes and Actinobacteria (Figure 1). Beside these groups, further well represented clades were Bacteroidetes and Acidobacteria. Of all sequences, 68% were classifiable at the family level, of which in turn 83% were also assignable to a genus. Overall, these sequences fell into 94 different genera and 73 families. Dominant phyla, families and genera are listed in Tab. 1 and the overall distribution including non-dominant phyla is presented in Figure 1. 

**Table 1:** Taxonomic distribution of sequencing reads into phyla and families, with their corresponding percentage and occurrence in chambers 1 (C1) and 2 (C2).

phylum		C1	C4	family		C1	C4	dominant genus
Firmicutes	32%	X	X	Bacillaceae 1	17%	X	X	*Bacillus*
				Paenibacillaceae	2%	X	X	*Paenibacillus*
				Bacillaceae 2	1%	X	X	
				Planococcaceae	1%	X	X	
Actinobacteria	27%	X	X	Propionibacteriaceae	3%	X	X	*Microlunatus*
				Conexibacteraceae	2%	X	X	*Conexibacter*
				Micrococcaceae	1%	X	X	*Arthrobacter*
				Microbacteriaceae	1%	X	X	*Agromyces*
				Nocardioidaceae	1%	X		
				Micromonosporaceae	1%	X	X	
				Geodermatophilaceae	1%	X	X	
				Solirubrobacteraceae	1%	X	X	*Solirubrobacter*
				Iamiaceae	1%	X	X	*Iamia*
				Rubrobacteraceae	1%	X	X	*Rubrobacter*
Proteobacteria	26%	X	X	Sphingobacteriaceae	3%	X		*Sphingomonas*
				Rhodospirillaceae	2%	X	X	*Skermella*
				Comamonadaceae	2%	X	X	*Diaphorobacter*
				Hyphomicrobiaceae	1%	X	X	
				Bradyrhizobiaceae	1%	X	X	
				Oxalobacteraceae	1%	X	X	*Massilia*
				Pseudomonadaceae	1%	X	X	*Pseudomonas*
Acidobacteria	9%	X	X	Gp16	5%	X	X	
				Gp6	2%	X	X	
				Gp4	1%	X	X	
Bacteroidetes	5%	X	X	Cytophagaceae	4%	X	X	*Adhaeribacter*
Chloroflexi	1%	X	X					

Further, the dominant genus within families are listed. Only phyla, families and genera with at least 1% overall read contribution are considered in the table.

### Gut bacteria

Adult honey bee guts have been screened both through high-throughput sequencing as well as cultivation methods for bacterial organisms [3,5,9,18]. It thus represents the most intensively studied honey bee associated microbiota with taxonomic and metagenomic information available. Although having a diverse set of gene sequences, microbiota of honey bee guts are reported to be of very low taxonomic diversity, i.e. only eight distinguishable taxa [3,5]. We were not able to identify seven of these, the only exceptions were organisms closely related to *Bartonella* spp. (designated as the alpha-1 group by Engel et al. [3]. 

This is in accordance with Martinson et al. [5] who were not able to diagnose any of the major gut bacteria of *Apis* in two Megachilidae bees related to *Osmia* (*Megachile odontostoma* and *Hoplitis biscutellae*). Further, they recognized a large proportion of Burkholderiales in several solitary bee families. In accordance, almost all of our β-proteobacterial sequences were assignable to the Burkholeriales. Two of our unique reads shared more than 97% sequence identity with *Burkholderia cepacia*, the most prominent species identified by Martinson et al. [5]. By 16S sequencing of isolates, Mohr and Tebbe [9] also found *B. capacia* to be present in *Osmia* larvae guts. We have however to account for the possibility that the finding of *Burkholderia cepacia* origins in contamination of plastic-ware used during DNA extraction, library preparation and sequencing [60]. As they were not found in the aforementioned samples originating from plants (but also other invertebrates) processed equally with the same methods and sequenced on the same chip, we assume this to be a biological signal and not an artificial finding. Despite that, *Burkholderia* contributed in our study only a minority to the Burkholderiales, whereas *Diaphorobacter, Massilia, Duganella* and *Variovorax* were more prominent genera. Burkholderiales function regarding *Osmia* is highly speculative: most originate from environmental sources as e.g. soil [61], but several are also known to interact as gut mutualists to arthropods by providing fitness benefits [62,63]. 

Beside the typical gut microbiota members of honey-bees, the G2III, C4 and M1 strains of *Bacillus subtilis* have been reported from their guts [64,65]. There they inhibit the growth of *Ascosphaera* spp. and *Paenibacillus* [64] and may thus have important roles in honey-bee immune defense. In our study, *Bacillus* was the overall most prominent genus contributing with 16% of reads to community structure. We found sequences with high similarity (>95%) to G2III and C4 of *Bacillus subtillis*, indicating that closely related bacteria with similar properties may be present within the gut of *Osmia bicornis* larvae. Similarly, *Bacillus circulans* was present in our samples which has been demonstrated to slow the growth of the honey-bee chalkbrood causative *Ascosphaera apis* [66].

*Enterobacter* and *Klebsiella* were also reported from guts of adult honey-bees [19], but not present within our samples. In general, only few Enterobacteriaceans were observable indicating that they might have only marginal importance in mason bee microbiota. 

We found several genera of the Acetobacteriaceae in our samples, i.e. *Acidimonas*, *Acidisphaera*, *Craurococcus*, *Saccharibacter* and *Tanticharoenia*. They have been identified to form symbiotic associations with insects, mostly within the midgut, but also other tissues [11,67]. Especially *Saccharibacter* and other genera are reported to have significant importance in food uptake and host survival [67]. 

Although the microbiota of honey-bees are very distinct, differences between internal and external honey-bee worker classes have been reported [5]. The presence of bacteria in eggs and larvae is currently under debate, however also the positive studies indicate that bacterial diversity is distinct and low of diversity [1,11,25]. Our results suggest a very different situation in solitary bees. The presence of gut bacteria within our larvae and the prominent differences in their composition to honey-bees may be results of unlike diets available during development. Honey-bee offspring is fed mostly by royal or worker jelly. By contrast, pollens are the primary source available to solitary mason bee larvae. Thus, gut bacteria may be essential to support *Osmia* larvae in their nutrient uptake, whilst honey-bees have developed an offspring nutrition system with eupeptic sources that is also efficient in the absence of bacteria or with a very limited set thereof. Further, a variety of gut bacteria seem to be present that may have importance in resistance against pathogens [3,8]. 

### Potential pathogens

We screened our samples for the most important bee specific bacterial pathogens, but also generalists broadly pathogenic to most arthropods. Most information about bee specific pathogens is again derived from honey-bees, whereas *Osmia* specific bacterial pathogens are currently unknown. Most prominent honey-bee pathogens belong to the Bacilli clade. This group was well represented in our data, accounting for 29% of our total sequences and thus dominating the Firmicutes phylum. Yet not all of these are pathogenic, a large number is considered to be intestinal as described above [65,68] or environmental non-pathogenic. Thus we restrict our implications to the well-known pathogenic organisms, i.e. *Bacillus cereus, Bacillus thuringiensis, Paenibacillus larvae* subsp. *larvae and Paenibacillus larvae* subsp. *pulvifaciens* [47]. 

In our samples, *B. thuringensis* strain CMBL-BT4 and *B. cereus* strains PDa-1 and IARI-B-24 were present with high confidence (> 97% sequence identity). Five other closely related strains with sequence identities above 95% were also observable, plus several further sequences with close relationships (>90% identity). Whereas *Bacillus cereus* is usually regarded as a pathogen [47], it has also been shown to be a non-pathogenic associate of three different solitary bees (*Centris flavofasciata*, *Crawfordapis luctuosa*, *Xyclocopa californica*) and may antagonize pathogenic *Paenibacillus* strains [59,65]. 

In total, 30 of our unique sequences matched *Paenibacillus larvae*, a phylum closely related to *Bacillus*. The group includes the severe honey-bee pathogenic *P. larvae* subsp. *larvae* and *P. larvae* subsp. *pulvifaciens* responsible for the American foulbrood. We did not find any of these pathogenic strains, and currently other subspecies are not known to be active threats for solitary bees. Sequence similarity was however above 90% in all cases and in one specific sequence with 99% for a *Paenibacillus larvae* not further distinguished in GenBank (GI: 125745150). The risks for *Osmia* to be affected by *Paenibacillus* is currently unknown, but it could partly contribute to the observed high larval mortality of *O. bicornis* (annual mean 11.8-28.3% in five study years) [16]. Infection risk could also be related to transmissions risk as well-connected *O. bicornis* populations showed higher larval mortality rates [16] and further seasonal temperatures, as germination has been demonstrated to be very slow below 30°C [69]. 

Thus, both *Bacillus* and *Paenibacillus* seem to be well represented in *Osmia* nests. Whether these include active strains pathogenic to *Osmia* is highly speculative. Yet, even if not posing a direct threat to mason-bees, it must be also considered that *Osmia* or their nests may also serve as an intermediate host, vector or habitat for bacteria that are virulent to honey bees.

A likely group of bacterial threats to *Osmia* are *Photorhabdus luminescens* and *Xenorhabdus nematophila* [51,52]. Both are nematode associated insect pathogens and are released by the vector after entering the haemocoele of insect larvae [70]. Death by toxic substrates and tissue disintegration through the bacteria occurs within 48 h. An nematode unrelated, but also larval specific insect pathogen is *Pseudomonas entomophila* dissolving the tissues and killing larvae with insecticidal toxins in similar time spans [49]. Read assignments for all three were found with > 97% sequence identity to reference sequences at GenBank. Thus, these three are very likely threats to *Osmia* and may also account for larval mortality.

*Clostridium botulinum*, producer of botulin toxins was found in honey bee colonies after death of worker bees [2]. It was not observable in our samples, although a variety of other *Clostridium* strains were present. Similarly, the major part of other remaining potential pathogens, i.e. *Melissococcus plutonius, Pseudomonas protegens, Rickettsiella grylli*, *Spiroplasma melliferum*, were not found at all within our samples. 

### Non-pathogenic intracellular bacteria

Several of the above mentioned gut bacteria and also some of the non-pathogenic strains of potential pathogenic species may play important roles in symbiotic interactions with the host. In addition to these, we screened for other bacteria reported to be intracellular within insect tissues. 

*Wolbachia*, as a non-lethal parasite affects the sex-ratio of offspring in many arthropods [55]. It is however also considered to have beneficial symbiotic activity in honey-bees [71]. It is widespread among insects, with high infection rates within populations and also reported for solitary bees [55,72]. Yet, *Wolbachia* was not present within our samples. *Mycoplasma, Rickettsia* and *Mesoplasma* are bacteria mediated by arthropods as vectors. The two latter are suspected to contribute to the host's vectorial aptitude by increasing its survival capability [48,71]. All three are known to be commensals of solitary bees, but were not found within our samples. Whether this is due to restricted sampling or presence exclusively in adult bees is highly speculative, however it has to be considered that inoculation occurs after leaving their nests.

The only other intracellular associates observable within our samples were two reads closely related to *Candidatus* Regiella *insecticola* . This species is reported to improve disease resistance against infectious fungi in aphids [53]. As the most important known pathogen to the mason-bee is a fungus, i.e. *Ascosphaera* spp. [17], antimycotic activity of this symbiont may contribute to host survival. 

### Flowers, pollen and nectar

Although a certain fraction of our sequences obtained from sequencing is assignable to *Osmia* by being directly positively or negatively associated bacteria, a large proportion is not accounted for by these explanations. These remaining species are mostly classifiable as environmental bacteria, collected by the bees and imported during their nest building, egg deposition and pollen storing activity. 

Interestingly, almost no Enterobactericeae were found, with exception of *Pantoea* and *Sodalis*. Enterbacteriaceae are reported to be a dominant bacterial group inhabiting flowers and nectar [73-75], so that we expected them to be present due to collection and deposition of pollens. A group also abundant on flowers and other plant tissues are Pseudomonadales [73,75], which were likewise only marginally observable within our samples (1% of total number of unique reads). Members of TM7 have as well been described as important bacterial member of flower microbiota [76], but were in our investigation observable only with negligible contribution to the overall community (1 sequence). *Deinococcus* reported in the same study was not present at all in both chambers [76]. Likewise, *Leuconostoc* and *Acinetobacter* reported by Álvarez-Pérez and Herrera [75] as well as Fridman et al. [77] from nectar were not at all and slightly present, respectively. This general low diversity of floral bacteria may be explainable by their chemical traits being strictly associated with sugars and other compounds present in flowers or other plant surfaces. Even if they were originally imported in high abundances during pollen collection in spring and early summer, they did not prevail until our sampling in fall. The lack of these species within the samples indicates that the nests are unsuitable habitats for these bacteria. An alternate explanation may be that active innoculation of pollens reduces abundance and diversity of pollen associated bacteria to promote conservation and digestibility, as known for honey bees [1,20,78] and other Hymenopterans [6]. This remains however speculative from our data and needs to be experimentally verified. 

### Soil

During their nest building activity, adult bees collect loam from the environment to separate the chambers from each other. Most of the remaining reads are likely to originate from this source. Closest relatives represented in the public sequence databases were in most cases isolates from forest grounds or other soils, as well as drainage and sludge systems or water sources. Thus, the major part of mason-bee nest microbiota seems to be only passively associated with the host itself, but rather a reflection of the microbial composition of the soil environment. As multiple trips are needed to close the walls, a diverse set of microbes may be incorporated. Home ranges are rather small compared with hive bees, indicating that the materials originate from nearby sites [79]. 

This diversity of microbial organisms is surprising as regulatory mechanisms to control environmental microbes are known for closely related species, also such with soil associated life cycles. European beewolfs apply Streptomycetes bacteria with antifungal properties to brood cell walls prior to oviposition [80]. Honey bee nest walls are reported to be treated with propolis to reduce microbial manifestations [81]. In our case, no antibacterial treatment seems to be present. The resulting soil bacteria diversity is very high and thus indicates a major difference to the antimicrobial and tended hives in which honey bee pupae grow up [25]. 

With our investigation, it is not possible to differentiate whether the microorganisms are active or by contrast in a dormant state. In the first case, the consequences are profound, opening new questions in solitary bee nutrition, habitat suitability, foraging, immunobiology and symbiotic interactions. It is currently unclear whether *Osmia* larvae have characteristics yielding tolerance against a diverse microbial environment, or on the contrary, whether an improved immunity and other benefits are induced by such a comprehensive set of bacteria. As has been shown for two Heteroptera species, environmental bacteria obtained from soil may provide beneficial effects for offspring fitness [63]. We thus speculate that *Osmia* larvae may gain symbiotic commensals through soil inoculating their brood cells, to improve their development.

## Conclusions

From a microbial perspective, *Osmia* females create brood cell microhabitats that resemble to a large part the surrounding environmental characteristics due to passive transport of bacteria from various sources into these nests. In contrast to plant-associated bacteria, those of soils seem to be able to flourish and dwell within the compartment walls between nest chambers. The resulting community is very diverse and may be composed of a patchwork from different collection sites. They may further be highly variable according to differences in soil characteristics of the bioregion and individual bee collection behavior.

From a bee perspective, the brood cell environment is very different from the controlled microbial system inhabited by honey bee colonies. This diversity of microorganisms may cause severe larval mortality and require specific physiological or symbiotic adaptations against microbial threats. They may however also profit from such a diverse environment through gain of mutualistic partners. These microbiological conditions require further attention as they are likely of great importance in bee offspring nutrition, development, and immune-response to multiple threats. We conclude that further studies of microbial interaction networks in solitary bees will help to explore so far unknown fitness components and will improve the understanding of driving factors of population dynamics.
